# Comparative Transcriptomic Analyses of Peripheral Blood Mononuclear Cells of COVID-19 Patients without Pneumonia and with Severe Pneumonia in the First Year of Follow-Up

**DOI:** 10.3390/v16081211

**Published:** 2024-07-28

**Authors:** Ozgecan Kayalar, Pelin Duru Cetinkaya, Vahap Eldem, Serap Argun Baris, Nurdan Kokturk, Selim Can Kuralay, Hadi Rajabi, Nur Konyalilar, Deniz Mortazavi, Seval Kubra Korkunc, Sinem Erkan, Gizem Tuşe Aksoy, Gul Eyikudamaci, Pelin Pinar Deniz, Oya Baydar Toprak, Pinar Yildiz Gulhan, Gulseren Sagcan, Neslihan Kose, Aysegul Tomruk Erdem, Fusun Fakili, Onder Ozturk, Ilknur Basyigit, Hasim Boyaci, Emel Azak, Tansu Ulukavak Ciftci, Ipek Kivilcim Oguzulgen, Hasan Selcuk Ozger, Pinar Aysert Yildiz, Ismail Hanta, Ozlem Ataoglu, Merve Ercelik, Caglar Cuhadaroglu, Hacer Kuzu Okur, Muge Meltem Tor, Esra Nurlu Temel, Seval Kul, Yıldız Tutuncu, Oya Itil, Hasan Bayram

**Affiliations:** 1 Koc University Research Center for Translational Medicine (KUTTAM), School of Medicine, Koc University, Istanbul 34010, Türkiye; hrajabi20@ku.edu.tr (H.R.); nkonyalilar18@ku.edu.tr (N.K.); dmortazavi18@ku.edu.tr (D.M.); korkuncsevalkubra@gmail.com (S.K.K.); sinem.erkann97@gmail.com (S.E.); gaksoy21@ku.edu.tr (G.T.A.); geyikudamaci20@ku.edu.tr (G.E.); habayram@ku.edu.tr (H.B.); 2 Department of Pulmonary Medicine, Faculty of Medicine, Cukurova University, Adana 01790, Türkiye; pelindurucetinkaya@hotmail.com (P.D.C.); pelinpinarw@hotmail.com (P.P.D.); oyabaydarr@yahoo.com.tr (O.B.T.); ismailhnt@gmail.com (I.H.); 3 Department of Biology, Science Faculty, Istanbul University, Istanbul 34134, Türkiye; vahap.eldem@istanbul.edu.tr (V.E.); selimcankuralay@ogr.iu.edu.tr (S.C.K.); 4 Department of Pulmonary Medicine, Faculty of Medicine, Kocaeli University, Kocaeli 41380, Türkiye; serapargun2002@yahoo.com (S.A.B.); ilknur.basyigit@gmail.com (I.B.); haboyaci@yahoo.com (H.B.); 5 Department of Pulmonary Medicine, Faculty of Medicine, Gazi University, Ankara 06500, Türkiye; kokturk.nurdan@gmail.com (N.K.); tansu.ciftci@gazi.edu.tr (T.U.C.); ikoguzulgen@gmail.com (I.K.O.); 6 Department of Pulmonary Medicine, Faculty of Medicine, Duzce University, Duzce 81620, Türkiye; pinaryildiz691@hotmail.com (P.Y.G.); dr.ozlemozturk@outlook.com (O.A.); evrem-33@hotmail.com (M.E.); 7 Department of Pulmonary Medicine, Altunizade Acibadem Hospital, Istanbul 34662, Türkiye; gulserensagcan@yahoo.com (G.S.); caglar.cuhadaroglu@acibadem.com.tr (C.C.); hacerkuzu@hotmail.com (H.K.O.); 8 Department of Pulmonary Medicine, Bilecik Training and Research Hospital, Bilecik 11230, Türkiye; neslihankose.nks@gmail.com; 9 Department of Pulmonary Medicine, Faculty of Medicine, Zonguldak Bulent Ecevit University, Zonguldak 67100, Türkiye; aysegultomruk@yahoo.com (A.T.E.); meltemtor@gmail.com (M.M.T.); 10 Department of Pulmonary Medicine, Faculty of Medicine, Gaziantep University, Gaziantep 27310, Türkiye; fusunfakili@yahoo.com; 11 Department of Pulmonary Medicine, Faculty of Medicine, Suleyman Demirel University, Isparta 32260, Türkiye; dronderozturk@gmail.com; 12 Department of Infectious Disease and Clinical Microbiology, Faculty of Medicine, Kocaeli University, Kocaeli 41380, Türkiye; emelazak@mynet.com; 13 Department of Infectious Diseases and Clinical Microbiology, Faculty of Medicine, Gazi University, Ankara 06500, Türkiye; hselcukozger@gazi.edu.tr (H.S.O.); pinar_aysert@yahoo.com (P.A.Y.); 14 Department of Infectious Diseases and Clinical Microbiology, Faculty of Medicine, Suleyman Demirel University, Isparta 32260, Türkiye; dresratemel@gmail.com; 15 Department of Biostatistics, Faculty of Medicine, Gaziantep University, Gaziantep 27310, Türkiye; sevalkul@gantep.edu.tr; 16 Department of Immunology, Koc University Research Center for Translational Medicine (KUTTAM), School of Medicine, Koc University Istanbul, Istanbul 34010, Türkiye; ytutuncu@ku.edu.tr; 17 Department of Pulmonary Medicine, Faculty of Medicine, Dokuz Eylul University, Izmir 35340, Türkiye; oya.itil@deu.edu.tr; 18 Department of Pulmonary Medicine, School of Medicine, Koc University, Istanbul 34010, Türkiye

**Keywords:** COVID-19, transcriptomic analysis, peripheral blood mononuclear cells (PBMC), long-term effects, inflammatory responses, pneumonia

## Abstract

The multisystemic effects of COVID-19 may continue for a longer time period following the acute phase, depending on the severity of the disease. However, long-term systemic transcriptomic changes associated with COVID-19 disease and the impact of disease severity are not fully understood. We aimed to investigate the impact of COVID-19 and its severity on transcriptomic alterations in peripheral blood mononuclear cells (PBMCs) following 1 year of the disease. PBMCs were isolated from the peripheral blood of healthy control donors who did not have COVID-19 (C; *n* = 13), from COVID-19 patients without pneumonia (NP; *n* = 11), and from COVID-19 patients with severe pneumonia (SP; *n* = 10) after 1-year of follow-up. Following RNA isolation from PBMCs, high-quality RNAs were sequenced after creating a library. Differentially expressed genes (DEGs) and differentially expressed long non-coding RNAs (DElncRNAs) were identified using Benjamini–Hochberg correction and they were analysed for hierarchical clustering and principal component analysis (PCA). Intergroup comparisons (C vs. NP, C vs. SP, and NP vs. SP) of DEGs and DElncRNAs were performed and hub genes were determined. Functional enrichment analyses of DEGs and DElncRNAs were made using Metascape (v3.5.20240101) and the first version of NCPATH. The RNA sequencing analysis revealed 4843 DEGs and 1056 DElncRNAs in “C vs. NP”, 1651 DEGs and 577 DElncRNAs in “C vs. SP”, and 954 DEGs and 148 DElncRNAs in “NP vs. SP”, with 291 DEGs and 70 DElncRNAs shared across all groups, respectively. We identified 14 hub genes from 291 DEGs, with functional enrichment analysis showing upregulated DEGs mainly linked to inflammation and osteoclast differentiation and downregulated DEGs to viral infections and immune responses. The analysis showed that 291 common and 14 hub genes were associated with pneumonia and that these genes could be regulated by the transcription factors JUN and NFκB1 carrying the NFκB binding site. We also revealed unique immune cell signatures across DEG categories indicating that the upregulated DEGs were associated with neutrophils and monocytes, while downregulated DEGs were associated with CD4 memory effector T cells. The comparative transcriptomic analysis of NP and SP groups with 52 gene signatures suggestive of IPF risk showed a lower risk of IPF in the SP group than the NP patients. Our findings suggest that COVID-19 may cause long term pathologies by modulating the expression of various DEGs, DeLncRNAs, and hub genes at the cellular level.

## 1. Introduction

The coronavirus disease 2019 (COVID-19), caused by severe acute respiratory syndrome coronavirus 2 (SARS-CoV-2), has evolved into a pandemic with high morbidity and mortality worldwide. According to the World Health Organization (WHO), there were 7,052,472 fatalities and 775,678,432 confirmed cases of COVID-19 worldwide as of 23 June 2024 [1]. The virus primarily affects the lungs but has also been reported to impact multiple organs and systems, including the brain, heart, and gastrointestinal system. Common symptoms such as fever, dry cough, headache, lethargy, myalgia, diarrhoea, and anosmia indicate COVID-19 infection in previously healthy individuals. COVID-19 often manifests as an asymptomatic or mild to moderate respiratory infection [2,3]. Additionally, the severity of the disease increases in patients with pre-existing co-morbidities such as obesity, respiratory, cardiovascular, and renal illnesses, which can rapidly progress into a severe life-threatening condition requiring immediate critical care support [4,5].

COVID-19 can cause acute, short-term, or long-term comorbidities. During the acute and sub-acute phases of the disease, respiratory problems such as sore throat, cough, dyspnoea, cardiac conditions including chest pain, palpitations, myocarditis, heart failure, and arrhythmia, and systemic complications such as hypercoagulation/thrombosis, leukopenia, and lymphopenia, which have been reported [6]. In terms of the mid- to long-term effects, more prolonged and persistent clinical pathologies including fatigue, headache, attention disorder, memory loss, hair loss, gastrointestinal distress, dyspnoea, anosmia, myocarditis, and fibrotic interstitial changes in the lungs have been reported [6,7,8,9,10].

The severity of COVID-19 is known to be associated with various risk factors, including age, severe hypoxemia, diabetes, heart diseases, hypertension, and chronic obstructive pulmonary disease (COPD), along with the presence of severe pneumonia [6]. Studies of COVID-19 have shown that the severity of the disease increases with accompanying pneumonia, leading to more severe and persistent long-term consequences [11,12]. In a study of children with COVID-19, the patients with severe pneumonia consistently had persistent symptoms [11]. An observational prospective study of 995 hospitalized patients with COVID-19 pneumonia showed that more than 40% of patients had at least one long-term COVID-19 symptom [13]. In another study, 95% of COVID-19 patients with moderate to severe pneumonia had persistent radiological changes in their lungs and 54% had fibrotic changes, following 4–7 months of recovery [14]. However, the study found no significant difference between the initial disease severity and the scores of radiological changes [14]. Although clinical and epidemiological studies on long-term COVID-19 symptoms have increased recently [15,16,17], long-term post-COVID complications and comorbidities are still poorly understood [18]. Furthermore, the mechanisms underlying the development of long-term complications in patients with severe COVID-19 or severe pneumonia are still not fully known.

Recent advances in omics studies and bioinformatics analysis have elucidated the mechanisms underlying many diseases, including COVID-19 [19,20]. Blood transcriptomic analysis contributes to understanding long-term or persistent transcriptional consequences after COVID-19 by analysing changes in the expression of free, vesicle-contained, and cellular-encoded and non-coding RNAs in the blood [21]. It is not clear whether there are any differences in the transcriptomes of peripheral blood mononuclear cells (PBMCs) of COVID-19 patients and whether the severity of the disease has any impact.

The main aim of our study was to investigate the impact of COVID-19 and its severity on transcriptomic alterations in PBMCs following 1 year of the disease. We collected PBMCs from healthy controls without SARS-CoV-2 infection, COVID-19 patients without pneumonia, and patients with severe pneumonia 1 year after the infection and performed unbiased next-generation RNA sequencing. We have demonstrated that COVID-19 can lead to differentially expressed genes (DEGs), differentially expressed long non-coding RNAs (DElncRNAs), and pneumonia-related hub genes that may play a role in the long-term pathologies as well as the fact that the severity of the disease may have an impact.

## 2. Material and Methods

### 2.1. Ethical Statement

This study followed the principles of the Declaration of Helsinki and was approved by the institutional ethics committee of Çukurova University School of Medicine (Approval number: 356/22.05.2021). Informed consent was obtained from the patients and the healthy volunteers for the collection of blood samples.

### 2.2. Data Availability

FASTQ sequences of the PBMC samples were deposited in NCBI Short Read Archive (SRA) under BioProject PRJNA895325.

### 2.3. Study Population

This study was conducted on patients who participated in the previously published TURCOVID study [22,23]. During the first wave of the COVID-19 pandemic (between 11 March and 18 July 2020), 1500 patients over the age of 18, who were monitored and treated because of COVID-19, were included in the targeted trial population in the multi-centre TTS-TURCOVID-19 registry cohort. A total of 831 patients were enrolled in the trial at 13 of the 26 locations (11 university hospitals, 2 sizable tertiary institutions, and 1 private hospital). A standard questionnaire was applied to current patients in the cohort over the phone after receiving written informed consent. Of the cohort of 831 patients, 272 (32.7%) could not be reached, 48 (5.8%) refused to participate in the study, 69 (8.3%) were excluded due to death, and the remaining 442 patients were included. Retrospective data entry was performed over the recorded files and an analysis of the medical records of 442 patients, who could be reached by phone, was applied. One year later, 138 patients from 11 centres who agreed to participate in the study and filled out a signed informed consent form were called for a follow-up check. A routine evaluation (clinical, laboratory, and radiological) of the patients was performed. Of these groups that were created, one group consisting of 13 cases (male: 8; female: 5) did not have radiological pneumonia when COVID-19 infection was detected and the other group of 14 cases (male: 10; female: 4) had clinically and radiologically severe pneumonia. The control group was composed of 13 subjects (male: 8; female: 5) who remained uninfected. Age, gender, and smoking status were taken into consideration to eliminate confounding factors in the randomization process. PBMCs were separated from the peripheral blood obtained from the patients and stored at −80 °C until they were used for RNA isolation. Following RNA quality assessment, samples from 2 patients without pneumonia and 4 patients with severe pneumonia were excluded from the study due to poor RNA quality. Thus, based on the RNA and overall quality analysis of PBMC samples, the number of cases in the three study groups was as follows: (i) patients without pneumonia during COVID-19 infection (no pneumonia, NP; *n* = 11), (ii) those with severe pneumonia during COVID-19 infection (severe pneumonia, SP; *n* = 10), and (iii) the healthy subjects with no disease (healthy controls, C; *n* = 13) (Table 1). When the long-term clinical symptoms of COVID-19 were analysed in the patient groups, of the 11 COVID-19 patients without pneumonia, 4 had long-term COVID-19 symptoms, whereas half of the 10 patients with severe pneumonia presented long-term COVID-19 symptoms and there was no significant difference between the two groups.

### 2.4. Blood Sample Collection and Isolation of Peripheral Blood Mononuclear Cells (PBMCs)

Approximately 20 mL of venous blood was collected from each participant and PBMCs were isolated using a solution of Lymphoprep™ (Alere Technologies, Oslo, Norway) by performing density gradient sedimentation at 2000 rpm for 20 min.

### 2.5. Transcriptome Library Construction and Next-Generation RNA Sequencing

Following isolation, the purity and integrity of RNA were assessed using a NanoDrop^TM^ spectrophotometer (Thermo Scientific, Waltham, MA, USA, Nanodrop 2000c) and RNA Nano 6000 Assay Kit of the Agilent Bioanalyzer 2100 system (Agilent Technologies, Santa Clara, CA, USA), respectively. Samples with RNA integrity number values > 7.5 were retained for further processing. Before library preparation, mRNA and long non-coding RNAs (lncRNAs) were enriched by the MGIEasy rRNA Depletion Kit (MGI Tech., Shenzhen, China) and DNase I treatment (NEB) was carried out for complete removal of the DNA according to the manufacturer’s instruction. RNA libraries were constructed from 500 ng of RNA using the MGIEasy RNA Library Prep Kit V3.0 protocol (MGI, Shenzen, China) according to the manufacturer’s instruction. Firstly, the RNA samples that had been enriched were fragmented using a fragmentation buffer. Subsequently, the resulting short fragments underwent reverse transcription and subsequent second-strand synthesis. The cDNA fragments were treated with standard library generation steps; end-repair, A-tailing, and adapter ligation. After purification with DNA clean beads, we enriched adapter-ligated fragments using 14 PCR cycles and subjected them to the following denaturation and single-strand circularization process to generate a single-stranded circular DNA library. These libraries were then used to generate DNA nanoballs (DNBs) by rolling circle replication (RCR). The resulting DNBs were then loaded into the patterned nanoarrays and the sequencing reaction was performed in a DNBSEQ-G400 sequencer with a pair-end read length of 100 bp.

### 2.6. RNA-Seq Data Analysis and Differential Expression Analysis

The quality of raw sequencing reads was checked with FastQC (Babraham Bioinformatics) before and after sequence trimming. For a comparison of the qualities of all RNA-Seq libraries, MultiQC software v1.19 was used to merge the results of FastQC [24]. Raw reads were filtered using fastp v0.23.0 to remove adaptor contamination, ambiguous (N > 5) bases, low-quality reads (Phred score, *Q* < 20), and fragments < 30 nt [25]. All other options used the default values. Summary statistics for RNA-Seq reads were computed using seqkit v2.0.0 [26]. Filtered reads were mapped to the human reference genome (GRCh38.p13, Ensembl Release 106) using Hisat2 v2.2.1 [27]. The alignment statistics were obtained with Sambamba v0.8.0 [28]. Count matrices and gene-level assignment were generated using featureCounts from Subread package v2.0.0 [29] with annotation version GRCh38.106 (Ensembl “.gtf”). Differential gene expression between groups was performed on raw counts using DESeq2 v1.34.0 after variance-stabilizing transform (vst) normalization [30]. Genes were considered as significantly differentially expressed if the adjusted *p*-value was less than 0.001 and log_2_FC > 1.0 (Benjamini–Hochberg (BH) multiple test correction method). Hierarchical clustering and principal component analysis (PCA) were performed using DEBrowser v1.20.0 to evaluate the correlation between control and disease samples [31]. Volcano plots of the most differentially expressed genes (DEGs) among comparison groups were generated using EnhancedVolcano v1.12.0 [32]. The top 34 upregulated and downregulated protein-coding genes (sorted by adjusted *p*-value in increasing order) for each comparison were selected for heatmap generation using the online tool ClustVis v2.0 [33]. The multiple plots generated from each condition were combined into one overall graph using the ggarrange() function available in the ggpubr R package v0.6.0 (https://rpkgs.datanovia.com/ggpubr/ last accessed on 1 January 2023).

### 2.7. Functional Annotation and Enrichment Analysis

Gene ontology (GO) and the Kyoto Encyclopedia of Genes and Genomes (KEGG) pathway enrichment analysis of DEG among all groups including control (C), no pneumonia (NP), and severe pneumonia (SP) was performed with Metascape using *Homo sapiens* (Ensembl Release 104) as background for enrichment [34]. The KEGG pathway enrichment analysis of DElncRNAs was performed for all groups (C, NP, and SP) with NCPATH using *Homo sapiens* (Ensembl Release 104) as background for enrichment [35]. The significance of enrichment analysis was estimated by Benjamini–Hochberg false discovery rate (FDR) < 0.05 correction. Protein–protein interaction (PIP) network analysis was performed using STRINGdb v12.0 [36]. Normalized expression data were used for the discovery of co-expressed modules, gene–disease interactions, interactions with transcription factors and their targets, and immune cell signatures using Metascape and Enrichr [37,38]. A variance filter value of 0.01 was used to ensure the highest level of statistical stringency and Pearson’s correlation method was selected for identification of the gene modules.

#### Identification of 52 Genes Associated with Idiopathic Pulmonary Fibrosis Risk in the PBMCs’ Transcriptome

In our PBMC transcriptome data, we compared up and down profiles based on the expression levels of 7 upregulated genes (PLBD1, TPST1, MCEMP1, IL1R2, HP, FLT3, and S100A12) and 45 downregulated genes (LCK, CAMK2D, NUP43, SLAMF7, LRRC39, ICOS, CD47, LBH, SH2D1A, CNOT6L, METTL8, ETS1, P2RY10, TRAT1, BTN3A1, LARP4, TC2N, GPR183, MORC4, STAT4, LPAR6, CPED1, DOCK10, ARHGAP5, HLA-DPA1, BIRC3, GPR174, CD28, UTRN, CD2, HLA-DPB1, ARL4C, BTN3A3, CXCR6, DYNC2LI1, BTN3A2, ITK, CD96, GBP4, S1PR1, NAP1L2, KLF12, IL7R, SNHG1, and C2orf27A) from a gene signature previously found to be predictive of IPF poor prognosis and COVID-19 outcome [39,40,41]. The genes for each comparison were selected for heatmap generation using the online tool ClustVis [33].

### 2.8. Statistical Analysis

Data were analysed for normality and continuous variables were compared using one-way variance analysis, ANOVA/Dunnett’s multiple comparison tests, or Kruskal–Wallis/Dunn’s multiple comparison tests in the context of the RNAseq count data based on gene expression analysis of the hub genes. The findings are presented as median ± interquartile (IQ) ranges or mean ± SD. *p* values were considered as significant if they were less than 0.05. PRISM version 8 (GraphPad Software Inc., San Diego, CA, USA) was used for the statistical analysis.

## 3. Results

### 3.1. Identification of DEGs and DElncRNAs among the Healthy Control, No Pneumonia, and Severe Pneumonia Groups

**Figure 1:**
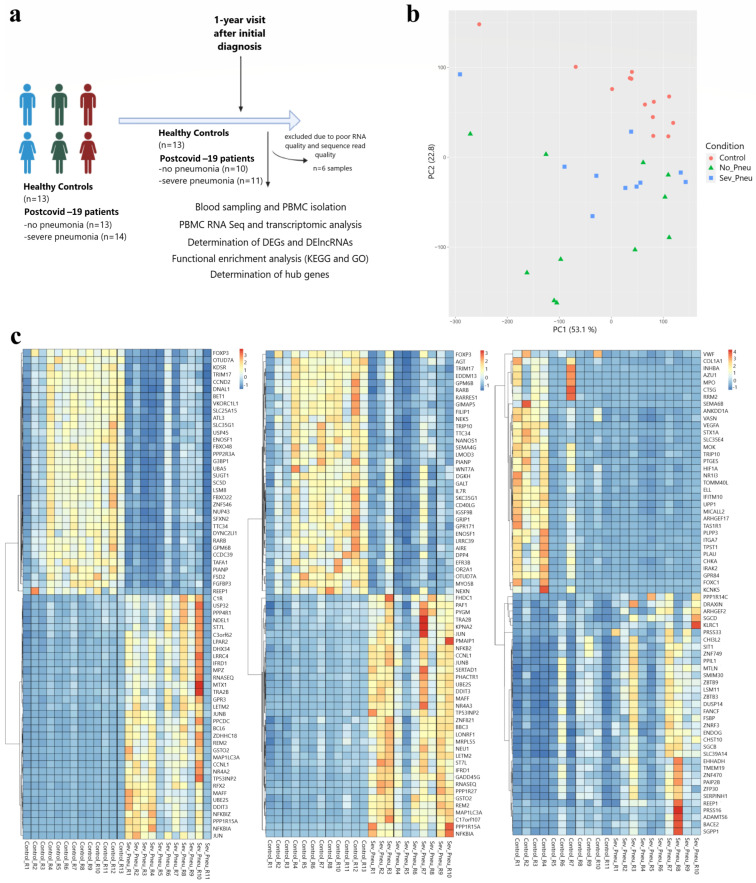
(**a**) A graphical abstract of the study is presented. (**b**) Principal component analysis is shown. Each point represents a sample. Colouring indicates groups. Red circles represent the control samples, green triangles represent the samples of patients with COVID-19 disease without pneumonia, and blue squares represent the samples of patients with COVID-19 disease and severe pneumonia confirmed by radiological data. (**c**) Heatmap of 34 upregulated and 34 downregulated DEGs in all group comparisons.

**Figure 2:**
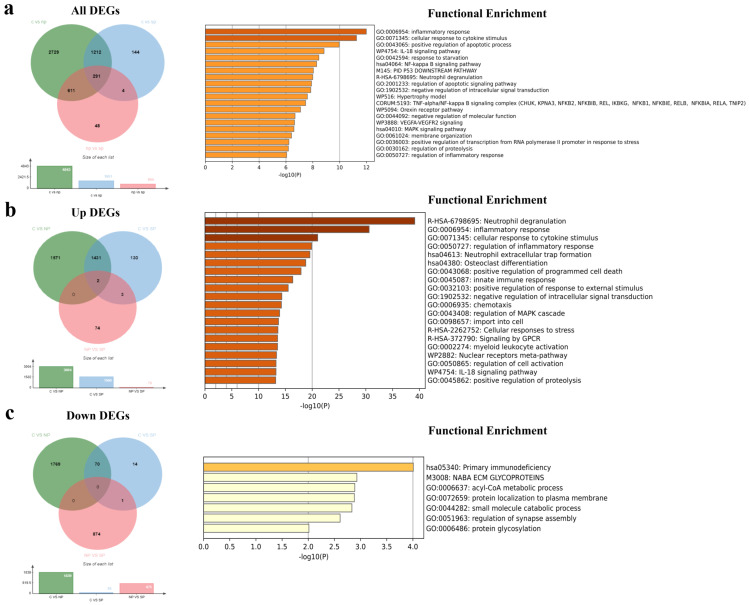
(**a**) All differentially expressed genes (DEGs) were compared, such as “C vs. NP”, “C vs. SP”, and “NP vs. SP, referring to the common 291 DEGs. Functional enrichment analysis (Kyoto Encyclopedia of Genes and Genomes (KEGG), Gene Ontology (GO), WikiPathways (WP), and the comprehensive resource of mammalian protein complexes (CORUM)) of the common 291 DEGs. (**b**) Upregulated DEGs among all comparisons and 1436 extended common upregulated genes. Functional enrichment analysis of common 1436 up-DEGs. (**c**) Downregulated DEGs among all comparisons and 71 extended common downregulated genes. Functional enrichment analysis of the common 71 down-DEGs. The green circle in the Venn diagram represents DEGs in the “C vs. NP” comparison, the blue circle represents DEGs in the “C vs. NP” comparison, and the red circle represents DEGs in the “NP vs. SP” comparison.

The success of the transcriptome sequencing reaction depends on the quality and quantity of the isolated RNA. Of the 40 PBMC-RNA samples sent for transcriptomic analysis, 36 samples with high-quality RNA were analysed, except for three patients with severe pneumonia and one patient without pneumonia. The RNA samples of the other 2 patients without pneumonia were excluded because of insufficient reading quality with sufficient number and accuracy for bioinformatic analysis. After the bioinformatic analysis of the raw data obtained after the sequence analysis, a total of 34 RNA samples from the C group (*n* = 13), the post-COVID-19 NP group (*n* = 11), and the post-COVID-19 SP group (*n* = 10) were included in the analysis (Figure 1a). The relative status of gene expression changes in all three groups is shown by PCA analysis using the transcriptomic gene expression profile. There was no significant difference in the gender and age distributions of the study groups. It was revealed that the C samples exhibited distinct clustering patterns compared to the other two groups. Also, the gene expression changes in individuals with NP and SP demonstrated greater disparities in comparison to the C group (Figure 1b). The group “C vs. NP” refers to genes that are up-regulated or down-regulated relative to the control group. “C vs. SP” also refers to genes that are upregulated or downregulated compared to the control. In the “NP vs. SP” group, it refers to the genes that are upregulated or downregulated in the SP group compared to the “NP” group. Moreover, a cluster heat map was created from 34 upregulated and 34 downregulated genes to show differential gene expression in each group (Figure 1c). In the comparison between C and NP, we identified 4843 DEGs including 3004 upregulated (up) genes and 1839 downregulated (down) genes as well as 1056 DElncRNAs including 694 up and 392 down lncRNAs (Figure 2a–c; Figure S1a–c). We identified 1651 DEGs including 1566 up and 85 down genes and 577 DElncRNAs including 493 up and 84 down lncRNAs between the C and SP (C vs. SP). In comparison, between NP and SP, 954 DEGs including 79 up and 875 down genes and 148 DElncRNAs including 5 up and 143 down lncRNAs have been identified (Figure 2a–c; Figure S1a–c). We then overlapped DEGs from all of the comparisons. Overall, we identified 291 DEGs and 70 DElncRNAs. Additionally, upregulated and downregulated DEGs and DElncRNAs were shown in Venn diagrams. Next, we performed functional enrichment analysis of common DEGs and DElncRNAs involved in severe COVID-19 after 1 year (Figure 2a–c; Figure S1a–c).

### 3.2. Functional Enrichment Analysis of All DEGs

To understand the function and pathways of all DEGs, enrichment analysis was performed. This analysis revealed that upregulated DEGs in the “C vs. NP” comparisons were involved in osteoclast differentiation (KEGG) and positive regulation of inflammatory process (GO:0050727). The downregulated DEGs, however, were associated with herpes simplex virus 1 infection (KEGG) and the positive regulation of natural killer cell-mediated immunity (GO:0002717). The upregulated DEGs were mainly located in tertiary granules and play a pivotal role in protein serine/threonine kinase activity. On the other hand, downregulated DEGs can be found in sarcoglycan and dystroglycan complexes and have a gene regulatory effect through binding to DNA via the RNA polymerase II transcription regulatory region sequence (Figure S2a). Upregulated DEGs in the “C vs. SP” comparisons were involved in osteoclast differentiation (KEGG) and the positively regulated inflammatory process (GO:0050727). Additionally, downregulated DEGs were associated with primary immunodeficiency (KEGG) and the alcohol catabolic process (GO:0046164). The upregulated DEGs were located in the secretory granule membrane and have cytokine receptor and G protein-coupled receptor activity. The majority of downregulated DEGs were found in keratin and intermediate filaments, acting as an acceptor for NAD or NADP in oxidoreductase activity (Figure S2b).

Upregulated DEGs in the “NP vs. SP” comparison were involved in herpes simplex virus 1 infection, viral myocarditis, arrhythmogenic right ventricular cardiomyopathy, sulphur metabolism, hypertrophic cardiomyopathy, and the TGF-beta signalling pathway (KEGG) in addition to the regulation of lymphocyte activation (GO:0051249) and some cardiac tissue morphogenesis processes (such as GO:0048738, GO:0061384, and GO:0051146). The downregulated DEGs in the comparison, however, were associated with transcriptional mis-regulation in cancer and TNF signaling (KEGG) as well as the positive regulation of transcription by RNA polymerase II (GO:0045944). The upregulated DEGs were mainly located in sarcoglycan and dystroglycan complexes and the main molecular function of these genes was found as serine-type endopeptidase activity. The location of downregulated DEGs were identified in specific and azurophil granules and they are involved in protein serine/threonine kinase activity (Figure S2c).

### 3.3. Functional Enrichment Analysis of Common DEGs and DElncRNAs

Enrichment analysis was conducted to gain a deeper understanding of the function and pathways of common DEGs. The results indicated that 291 common DEGs across all DEGs were primarily engaged in inflammatory response processes, including TNF-α, NF-κB, and MAPK signalling pathways (Figure 2a). Moreover, the extended common 1436 DEGs in upregulated DEGs were involved in neutrophil degranulation, neutrophil extracellular trap formation, osteoclast differentiation (KEGG), and the cellular inflammatory response (GO:0006954 and GO:0071345) (Figure 2b). Furthermore, the extended common 71 DEGs in downregulated DEGs were mainly elaborated in primary immunodeficiency (KEGG) and some metabolic processes such as the acyl-CoA process (GO:0006637), small molecule catabolic process (GO:0044282), and protein glycolisation (GO:0006486) (Figure 2c). The results indicated that 70 common lncRNAs across all DElncRNAs were principally engaged in MAPK and Rap1 signaling pathways (KEGG). Moreover, the extended common 457 lncRNAs in upregulated DElncRNAs played a role in the MAPK signaling pathway, focal adhesion, cell cycle, and insulin resistance (KEGG). Furthermore, the extended common 79 lncRNAs in downregulated DElncRNAs were mainly involved in thermogenesis and the mTOR signaling pathway (KEGG). Finally, our data have revealed that 23 common DEGs and 70 DElncRNAs were enriched in MAPK, Rap1, and AMPK signaling pathways (KEGG) (Table S1).

### 3.4. Identification of Hub Genes via the Protein–Protein Interaction (PPI) Network

**Figure 3:**
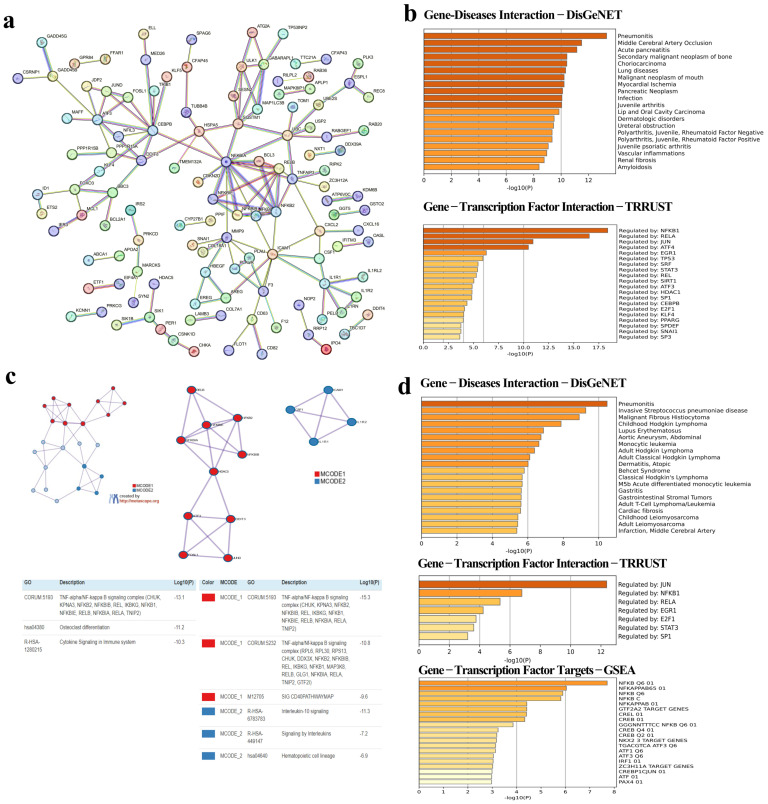
(**a**) Protein–protein interaction analysis (PPI) of 291 common genes were determined as a result of the comparison of DEGs in all comparable groups. The nodes indicate proteins, and edges indicate the number of interactions. (**b**) Gene–disease (DisGeNET) and gene–transcription factor interactions (TRRUST) of these genes. (**c**) PPI analysis of hub genes from 291 genes. (**d**) Gene–diseases (DisGeNET), gene–transcription factor interactions (TRRUST), and gene–transcription factor targets (GSEA) of these hub genes. The graph shows the diseases, transcription factors, and their targets with which DEGs are highly correlated in these intergroup comparisons. The vertical axis shows the disease names obtained from the DisGenet database and the horizontal axis shows the −log10 (P) statistical significance level.

The PPI network analysis revealed an association of DEGs among C, NP, and SP groups. We identified 291 common DEGs consisting of 291 nodes with 142 edges (Figure 3a). In total, 36 genes were determined from the 291 common genes using Metascape (v3.5.20240101) online software. Among the 36 genes, the highest scoring (Interactions ≥ 3) in 23 central genes, namely *ICAM1*, *TUBB4B*, *MARCKS*, *NFKB2*, *NFKBIA*, *NFKBIE*, *HDAC5*, *ATF3*, *DDIT3*, *F3*, *PRKCD*, *IL1R1*, *AREG*, *CSF1*, *IL1R2*, *TOM1*, *RAB11FIP1*, *FSCN1*, *ULK1*, *RELB*, *NFKBIB*, *FOSL1*, and *JUND*, were identified from the PPI network in combination with the Metascape (Table 2). When we analysed these 23 genes, we obtained a hub gene network with a total of 14 genes in 2 modules (Figure 3c) and then, we revealed that these genes were specifically involved in the TNF-α/NF-κB signaling complex, osteoclast differentiation, and cytokine signaling in the immune system.

### 3.5. Identification of Gene–Disease, Gene–Transcription Factor, and Gene–Transcription Factor Target Interactions

The gene–disease interaction via DisGeNET interestingly showed that the 291 common genes related to pneumonitis, middle cerebral artery occlusion, acute pancreatitis, secondary malignant neoplasm of bone, choriocarcinoma, lung diseases, oral malignant neoplasm, myocardial ischemia, pancreatic neoplasm, infections, and juvenile arthritis. We identified that these genes were mostly regulated by the transcription factors NFKB1, RELA, JUN, and ATF4 (Figure 3b). Interestingly, DisGeNET analysis have confirmed that 14 identified hub genes were primarily related to pneumonitis. Further analysis has illustrated that JUN can regulate above-mentioned genes, especially those carrying NFKB Q6, one of the transcription factor targets (Figure 3d).

### 3.6. Identification of Immune Cell Type Signatures of the Common and Central DEGs

**Figure 4:**
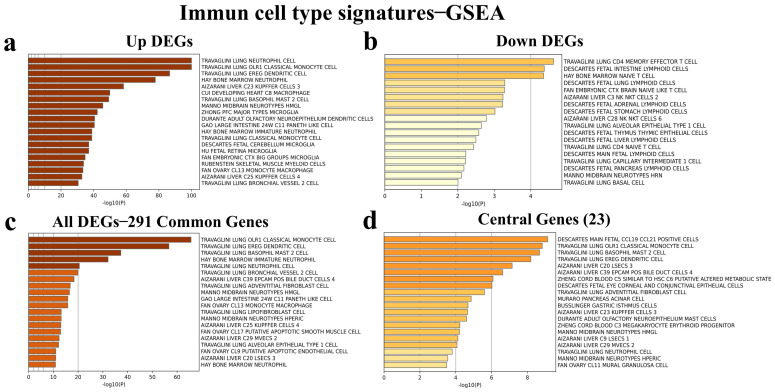
Immune cell signatures of (**a**) the extended common upregulated DEGs, (**b**) the extended common downregulated DEGs, (**c**) the 291 common DEGs, and (**d**) the 14 hub genes. The graph shows the immune cell signatures with which DEGs are highly correlated in these intergroup comparisons. The vertical axis shows the immune cell names obtained from the databases and the horizontal axis shows the −log10 (P) statistical significance level.

Metascape analysis was performed in order to determine immune cell type signatures in the extended common 1436 upregulated, the extended common 71 downregulated, 291 common, and 23 central DEGs; the results showed that the 1436 DEGs could regulate neutrophils and monocytes (Figure 4a), downregulated common 71 DEGs regulate CD4 memory effector T cells (Figure 4b), and common 291 DEGs govern monocytes, dendritic cells, basophils, and neutrophils (Figure 4c). Interestingly, 23 central genes mainly regulated CCL19 and CCL21 positive cells, monocytes, basophils, and dendritic cells (Figure 4d).

### 3.7. Gene Expression Analysis Based on RNA-seq Count Data of 14 Hub Genes

**Figure 5:**
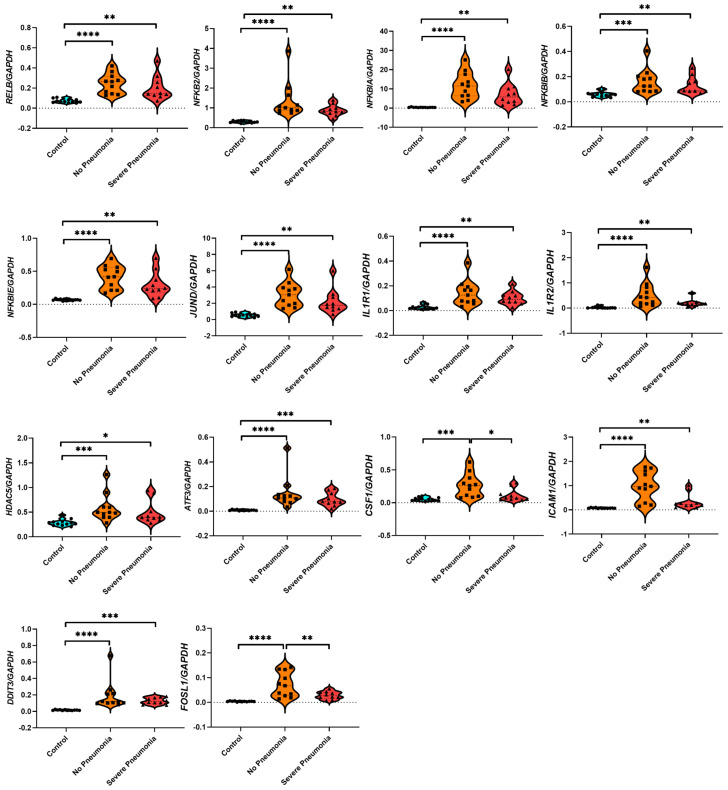
Gene expression analysis based on RNA-seq count data of 14 hub genes. All target genes’ counts were normalized to the counts of the individual GAPDH. Blue circles show control individuals, orange and squares show the individuals with no pneumonia, and red and triangles show the individuals with severe pneumonia. Statistical significance is shown as * *p* < 0.05, ** *p* < 0.01, *** *p* < 0.001, and **** *p* < 0.0001.

We performed a gene expression analysis based on statistical analysis of the normalized counts of the hub genes and the GAPDH of each patient and control. According to these results, all of the genes were significantly upregulated in the NP groups compared to healthy control individuals. Also, the other genes except *FOSL1* and *CSF1* were significantly upregulated in the SP group compared to the control groups. The *FOSL1* (*p* < 0.05) and *CSF1* (*p* < 0.01) genes were downregulated in the SP group compared to the NP group. There were no significant differences among the other genes in comparison to the NP and SP groups (Figure 5).

### 3.8. Investigation of 52 Idiopathic Pulmonary Fibrosis (IPF)-Related Gene Signatures in the PBMC Transcriptome of Patients with Non-Pneumonia and Severe Pneumonia

To identify idiopathic pulmonary fibrosis (IPF)-related genes, the transcriptome signals of the PBMCs from “NP“ and “SP” groups were analysed using a predefined set of 52 PBMC signature genes; results were visualized in a heatmap. As a result, the expression levels of PLBD1, TPST1, MCEMP1, IL1R2, HP, FLT3, and S100A12, which were low in the control group, significantly increased NP groups. Moreover, PLBD1, S100A12, and MCEMP1 gene expression increased in SP groups, while no significant changes were noted in the other four genes (Figure S1d). Also, we demonstrated that the expression levels of the genes, which were high in the control group, significantly decreased in both the NP and SP groups (Figure S1d). The 52 gene list is in Figure S2 in the Supplementary Materials.

## 4. Discussion

In the present study, we conducted a comprehensive analysis of RNA sequences from PBMCs to interrogate the differential gene and lncRNA expression profiles amongst healthy controls, COVID-19 patients without pneumonia (NP), and with severe pneumonia (SP). We identified 14 hub genes from 291 DEGs, mainly linked to inflammation and osteoclast differentiation, or to viral infections and immune responses. Furthermore, 291 common and 14 hub genes were associated with pneumonia and various unique immune cell signatures across DEG categories were associated with neutrophils, monocytes, and CD4 memory effector T cells. These findings suggest that COVID-19 may lead to persistent expression of various DEGs, DeLncRNAs, and hub genes, which may play a role in long-term pathologies, in which the disease severity may have an impact.

The analyses demonstrated that the signals of TNF-α/NFκB and MAPK were high in the PBMC transcriptome in the presence of pneumonia. This condition persisted for 1 year after severe COVID-19, which could have an impact on the regulation of inflammatory and fibrotic processes that these signals could trigger. Such modulation and associated signatures with neutrophils might be the underlying reason for the problems signaled in patients. In one of the pioneering studies, proteomic analysis was conducted with blood plasma collected for two 6-month visits and multiplex gene expression analysis was performed with RNA obtained from nasal epithelial cells. This study showed the presence of elevated systemic inflammatory signals even 3–6 months post-disease and reported that the radiological and functional alterations observed in these individuals did not revert to normal within a 12-month [42]. It has been shown that these findings are associated with increased inflammatory signals and interstitial changes in the lungs after COVID-19 [42]. These findings corroborate the alterations observed in our studies. However, our investigation is distinguished as the inaugural transcriptomic analysis conducted with PBMCs isolated from blood samples obtained at the end of one year from randomly selected severe COVID-19 patients with and without severe pneumonias as well as from control subjects matched for age and gender. The PCA analysis and cluster heat maps provided a clear visual representation of the variations in gene expression, with the control samples distinctly separating from the patient groups. This difference indicates a fundamental shift in gene expression dynamics after COVID-19, which could help in understanding the disease’s lingering effects and potential avenues for therapeutic interventions.

The identification of DEGs revealed distinct molecular signatures between the groups. Based on group comparisons of C vs. NP, C vs. SP, and NP vs. SP, DEGs that were upregulated in both NP and SP patients compared to C individuals were enriched in osteoclast differentiation and HIF1α, IL-17, and TNF-α signaling pathways. At the same time, downregulated genes were enriched in Herpes simplex virus 1 infection in the “C vs. NP” comparison and primary immunodeficiency in the “C vs. SP” comparison. This situation suggests the long-lasting effect observed following excessively increased inflammatory signals after virus infection. According to the NP group, it was determined that the upregulated genes in patients with SP observed in their radiology and clinics were mainly clustered in herpes simplex virus 1 infection and cardiac changes secondary to the virus. Most of the downregulated genes were, in turn, involved in the transcriptional mis-regulation in cancer and the downregulation in TNF-α signalling. The comparison of “NP vs. SP” revealed unique DEGs that played a role in cardiac processes and viral infections. This transcriptomic change may suggest an increased propensity for cardiovascular involvement and increased susceptibility to viral infections in patients with severe COVID-19. Previous studies demonstrated that TNF-α can accelerate osteoclast differentiation independently through the receptor activator of NF-κB ligand (RANKL)/RANK/TRAF6 pathway, while IL-17A supports this process by acting through the RNAKL-JNK1 signaling pathway [43,44,45]. Research has shown that the differentiation of osteoclasts is tightly regulated by the binding of macrophage colony-stimulating factor (M-CSF) and RANKL to respective receptors on the surface of osteoclast precursor cells. This regulation is mediated by M-CSF, which stimulates osteoclast proliferation, and RANKL inducing osteoclast differentiation [46].

In a murine model study, it has been proven that silica initiates osteoclast-like differentiation in silica-induced pulmonary fibrosis, recruiting monocytes to the tissue. Differentiation of alveolar macrophages is mediated by the osteoclastogenic cytokine RANKL, released from pulmonary lymphocytes and type II alveolar epithelial cells [47]. Collectively, these studies suggest the potential involvement of this mechanism in the persistent effects observed post-viral infection.

The identification of common DEGs and DElncRNAs across all comparisons, as well as the elucidation of hub genes, provided a holistic view of the pivotal molecular players in a 1-year period after severe COVID-19 conditions. In the present study, the enrichment of these common DEGs in inflammatory response processes and signaling pathways, such as TNF-α, NF-κB, and MAPK, highlights the long-term transcriptional changes associated with the persistent inflammatory state and complications in severe COVID-19 patients. In a previous study, which used the PBMC transcriptomic approach, 12 novel genes that were associated with short- and long-term organ dysfunction and COVID-19 deaths in COVID-19 patients were identified. These genes were different from those we identified in the present study [48,49].

Our PPI network analysis revealed 14 hub genes, namely *RELB*, *NFKB2*, *NFKBIA*, *NFKBIB*, *NFKBIE*, *HDAC5*, *ATF3*, *DDIT3*, *FOSL1*, *JUND*, *CSF1*, *ICAM1*, *IL1R1* and *IL1R2*, and their interconnectedness. These hub genes were identified to be of critical involvement in immune responses and inflammation, hence indicating that the possible outcome of post-acute COVID-19 conditions may result from continued immune system activation and possible dysregulation. Multi-omics analysis performed on pulmonary tissues of COVID-19 patients [47] has revealed that the mechanisms of senescence, inflammation, apoptosis, coagulation, and fibrosis are enhanced and characterize the complications observed at the early stage of the disease [50]. Furthermore, the genes and proteins playing a role in these mechanisms and their associated signaling networks were elucidated. Intriguingly, within these networks, certain molecular entities (*RELB*, *DDIT3*, and *FOSL1*) were identified as hub genes with increased expression in our patient samples during the 1-year follow-up after severe COVID-19. These genes are believed to bridge the networks of apoptosis, inflammation, and fibrosis [50]. Hovewer, what is interesting to note is that the upregulation of these genes continued throughout the entire 1-year recovery period from severe COVID-19, starting from the acute phase. They may contribute to the development of outcomes related to inflammatory and fibrotic complications after severe COVID-19 accompanied by pneumonia.

Our gene–disease interaction analysis provided intriguing links between the common DEGs and various diseases, especially including pneumonitis, other lung-related conditions, and arthritis. Transcriptomic study of the acute phase PBMCs of COVID-19 patients revealed several immune-related illnesses, such as pneumonia, arthritis, and septicaemia, in earlier research [51]. As known, SARS-CoV-2 can spread in the lower respiratory tract of severe patients, resulting in hypoxemia, severe pneumonia, and acute respiratory distress syndrome [52]. Pathological samples from the lungs of patients suffering from COVID-19 during the acute phase showed intense inflammation of cellular markers linked to apoptosis-dependent fibrotic alterations. It has been postulated that these signals might be connected to inflammation and fibrotic changes elicited in acute pneumonia [50].

The immune cell-type signature analyses showed that, between the upregulated and downregulated DEGs, there were apparent differences in the profiles. Neutrophils and monocytes were dominant among the upregulated DEGs, while CD4 memory effector T-cells were dominant among the downregulated DEGs. Transcriptomic analyses of PBMCs from patients with acute COVID-19 showed that upregulated genes were enriched in bone marrow, blood CD33+ myeloid cells, and CD14+ monocytes. On the contrary, downregulated genes were enriched for CD4 and CD8 T-cells, respectively [51]. In such a signature of immunity cell types, alterations could be due to the immune response of the patients and their susceptibility to secondary infections in long-term COVID-19. Among the hub genes, the analysis of gene expression among the groups revealed a notable increase in the NP group compared to the C group. The observed increase in gene expression of these genes in the NP group and the different expression trends in the SP group indicate a complicated interaction of molecular mechanisms that contribute to the diverse clinical outcomes observed in long-term COVID-19 patients. The investigation of IPF-related gene signatures revealed a subset of genes with altered expression in the NP and SP groups, providing a potential link between COVID-19 and the risk of developing IPF-like conditions. Of the 52 well-described IPF signature genes in our study, only 7 were upregulated in COVID-19 patients with NP, whereas in the SP group, only few genes, namely *PLBD1*, *S100A12*, and *MCEMP1*, were upregulated. The upregulation of seven genes correlated with severe IPF and COVID-19 outcomes is evidenced by previous studies [39,40,41]. Furthermore, all the 45 genes are shown to be expressed with a downward trajectory in the two groups of patients and previously connected to the risks of severe IPF and COVID-19 [41]. The NP group showed a similar signature with IPF. The high-risk genes that were identified in most of these high-risk genes in CD14+ monocytes, along with dendritic cells and neutrophils with different cell groups, together seem to be pointing toward the critical regulator of the mediated response. Studies have shown that the CSF1 gene is among the genes that play an executive role in bleomycin-induced lung fibrosis. It has been observed that lung fibrosis is prevented in the deficiency of this gene [53]. There is also increasing evidence that the receptor inhibition of this gene is a potential treatment approach for lung fibrosis [53,54,55]. Based on our gene expression analysis, all genes except *CSF1* were higher in patients who had COVID-19, regardless of COVID-19 severity. While the CSF1 gene increased in the NP group, it showed a significant decrease in severe COVID-19 patients. This may explain why fibrotic signals were observed less in the SP group than in the NP group.

The main limitations of our study were, however, the small number of samples, which did not allow us to perform correlation and regression analyses of hub genes determined with patients’ clinical data. It would also be informative to determine the biomarker potential of these genes by testing them in a validation cohort, which was not possible in the current study.

## 5. Conclusions

Our study is the first to reveal the PBMC gene signature associated with transcriptomic changes in the 1-year period after severe COVID-19. It has provided a comprehensive transcriptomic analysis of PBMCs, projecting DEGs, DElncRNAs, and pneumonia-related hub genes that may be involved in long-term effects after severe COVID-19. However, there is a need to assess the persistent inflammatory status and changes in immune cell type signatures in the follow-up of COVID-19 patients for longer time periods that can be objectives of future research. Furthermore, functional studies with larger cohorts focusing on longer-term effects are needed to validate pneumonia-associated hub genes, which we identified in the current study.

## Figures and Tables

**Table 1:** Demographics of the study subjects.

Groups	Healthy Controls (*n* = 13)	No Pneumonia (*n* = 11)	Severe Pneumonia (*n* = 10)
Gender * (F/M)	5/8	4/7	3/7
Age **	45 (41.5–52)	56 (41–65)	47 (45.5–65.25)

* Gender: Female (F) and Male (M), ** Age data were demonstrated as the median (Interquartile Range (IQR)).

**Table 2:** Following the comparative analysis of all of the up-regulated and down-regulated genes across the control, no pneumonia, and severe pneumonia groups, 36 hub genes were identified from a total of 291 common DEGs. Subsequently, a selection of 23 central genes, each interacting with 3 or more other genes, was made, along with an investigation into the genes that they interact with.

Gene Symbol	Number of Genes Interacted	Names of Genes Interacted
*ICAM1*	6	*AREG*, *CSF1*, *IL1R1*, *IL1R2*, *PRKCD*, *TUBB4B*
*MARCKS*	6	*F3*, *FSCN1*, *PRKCD*, *RAB11FIP1*, *TUBB4B*, *ULK1*
*TUBB4B*	6	*F3*, *GABARAPL1*, *ICAM1*, *MARCKS*, *SNAI1*, *ULK1*
*HDAC5*	5	*ATF3*, *DDIT3*, *NFKB2*, *NFKBIA*, *NFKBIE*
*NFKBIE*	5	*HDAC5*, *NFKB2*, *NFKBIA*, *NFKBIB*, *RELB*
*NFKB2*	5	*HDAC5*, *NFKBIA*, *NFKBIB*, *NFKBIE*, *RELB*
*NFKBIA*	5	*HDAC5*, *NFKBIB*, *NFKBIB2*, *NFKBIE*, *RELB*
*ATF3*	4	*DDIT3*, *FOSL1*, *HDAC5*, *JUND*
*F3*	4	*ICAM1*, *MARCKS*, *PRKCD*, *TUBB4B*
*DDIT3*	4	*ATF3*, *FOSL1*, *HDAC5*, *JUND*
*IL1R1*	4	*CSF1*, *ICAM1*, *IL1R2*, *TOM1*
*PRKCD*	4	*F3*, *FSCN1*, *ICAM1*, *MARCKS*
*AREG*	3	*ERG*, *HBEGF*, *ICAM1*
*CSF1*	3	*ICAM1*, *IL1R1*, *IL1R2*
*FOSL1*	3	*ATF3*, *DDIT3*, *JUND*
*FSCN1*	3	*MARCKS*, *MCL1*, *PRKCD*
*IL1R2*	3	*CSF1*, *ICAM1*, *IL1R1*
*JUND*	3	*ATF3*, *DDIT3*, *FOSL1*
*NFKBIB*	3	*NFKB2*, *NFKBIA*, *NFKBIE*
*RAB11FIP1*	3	*IFITM3*, *MARCKS*, *TOM1*
*RELB*	3	*NFKB2*, *NFKBIA*, *NFKBIE*
*TOM1*	3	*IL1R1*, *IFITM3*, *RAB11FIP1*
*ULK1*	3	*GABARAPL1*, *MARCKS*, *TUBB4B*
*ERG*	2	*AREG HBEGF*
*GABARAPL1*	2	*TUBB4B*, *ULK1*
*HBEGF*	2	*AREG*, *ERG*
*IFITM3*	2	*RAB11FIP1*, *TOM1*
*MCL1*	2	*FSCN1*, *USP2*
*SNAI1*	2	*TUBB4B*, *USP2*
*USP2*	2	*MCL1*, *SNAI1*
*GADD45B*	2	*GADD45G*, *MAP2K3*
*GADD45G*	2	*GADD45B*, *MAP2K3*
*MAP2K3*	2	*GADD45B*, *GADD45G*
*CSNK1D*	2	*HSPA5*, *NOP2*
*HSPA5*	2	*CSNK1D*, *NOP2*
*NOP2*	2	*CSNK1D*, *HSPA5*

## Data Availability

Data are contained within the article and Supplementary Materials. The raw data have been deposited in NCBI Short Read Archive (SRA, https://www.ncbi.nlm.nih.gov/sra, accessed on 19 April 2024) under BioProject PRJNA895325.

## Supplementary Materials

The following supporting information can be downloaded at: https://www.mdpi.com/article/10.3390/v16081211/s1, Figure S1: (a) All DElncRNAs comparison among “C vs. NP”, “C vs. SP” and “NP vs. SP”, and common 70 DElncRNAs. Functional enrichment analysis (KEGG) of common 70 DElncRNAs. (b) Upregulated DEGs among all comparisons, and 457 extended common upregulated lncRNAs. Functional enrichment analysis of common 457 upregulated lncRNAs. (c) Downregulated DEGs among all comparisons, and 79 extended common downregulated lncRNAs. Functional enrichment analysis of common 79 downregulated lncRNAs The green circle in Venn diagram represents DElncRNAs in “C vs. NP” comparison, and blue circle represents DElncRNAs in “C vs. NP” comparison, and red circle represents DElncRNAs in “NP vs. SP”comparison; Figure S2: (a) KEGG and GO terms of upregulated and downregulated DEGs between Healthy control and No pneumonia groups. (b) KEGG and GO terms of upregulated and downregulated DEGs between Healthy control and Severe pneumonia groups. (c) KEGG and GO terms of upregulated and downregulated DEGs between No pneumonia and Severe pneumonia groups. The top 10 functional terms are sorted from the highest statistical significance score in light color to the lowest score in dark color. (d). Evaluation of 52 validated PBMC genes for disease progression risk assessment in patients with IPF and COVID-19 in post-COVID-19 patients; Table S1: Top 10 functional enrichment (KEGG) analysis of upregulated, downregulated, common, and hub genes. Data were obtained from NCPATH database.
