# Supplementary material for: Comparative Transcriptomic Analyses of Peripheral Blood Mononuclear Cells of COVID-19 Patients without Pneumonia and with Severe Pneumonia in the First Year of Follow-Up
Source: Viruses. 2024 Jul 28;16(8):1211. doi: 10.3390/v16081211 (PMC11358892; doi:10.3390/v16081211)
Supplement: Supplementary file 1 [file viruses-16-01211-s001.zip › Supplementary Table S1.pdf]

Table S1. Top 10 functional enrichment (KEGG) analysis of upregulated, downregulated, common, and hub genes.

**Common 70 DElncRNAs-KEGG Functional Enrichment Analysis**				
**hsaid**	**pathway**	***p*-adjust**	***p*-value**	**genes**
hsa04010	MAPK signaling pathway	9.53933919587235e-17	1.1776961970212778e-18	223
hsa04015	Rap1 signaling pathway	2.444343597210432e-12	9.053124434112712e-14	165
hsa05215	Prostate cancer	2.444343597210432e-12	8.804392672203224e-14	84
hsa04152	AMPK signaling pathway	2.6608061331489008e-12	1.3139783373574818e-13	101
hsa04722	Neurotrophin signaling pathway	9.495107472233918e-12	5.861177451996246e-13	98
hsa04520	Adherens junction	1.0647390579295397e-11	7.886955984663257e-13	65
hsa05165	Human papillomavirus infection	1.7895532145452728e-11	1.5465274693601124e-12	242
hsa04218	Cellular senescence	2.324204209971124e-11	2.295510330835678e-12	124
hsa01522	Endocrine resistance	2.6330695483170815e-11	2.925632831463424e-12	82
hsa04510	Focal adhesion	2.6692401663290662e-11	3.2953582300358844e-12	151
**Upregulated common 457 DElncRNAs-KEGG Functional Enrichment Analysis**				
**hsaid**	**pathway**	***p*-adjust**	***p*-value**	**genes**
hsa04010	MAPK signaling pathway	3.805507722682358e-15	5.0740102969098097e-17	250
hsa04510	Focal adhesion	2.892857382144406e-11	7.714286352385083e-13	173
hsa04110	Cell cycle	1.5296857804112035e-10	6.118743121644814e-12	113
hsa04931	Insulin resistance	3.8773516016758865e-10	2.0679208542271393e-11	99
hsa04152	AMPK signaling pathway	3.937591631844922e-10	2.6250610878966148e-11	109
hsa05215	Prostate cancer	6.763741254546563e-10	5.41099300363725e-11	89
hsa04722	Neurotrophin signaling pathway	8.295350701461146e-10	7.742327321363737e-11	106
hsa04015	Rap1 signaling pathway	1.3129177372101546e-09	1.4004455863574982e-10	181
hsa04150	mTOR signaling pathway	1.5725927540822539e-09	1.8871113048987045e-10	132
hsa04218	Cellular senescence	2.2339661496262503e-09	3.490933500542371e-10	136
**Downregulated common 79 DElncRNAs-KEGG Functional Enrichment Analysis**				
**hsaid**	**pathway**	***p*-adjust**	***p*-value**	genes
hsa01524	Platinum drug resistance	0.00024072411175947426	1.2269490024761625e-05	35
hsa04714	Thermogenesis	0.00024072411175947426	1.4589340106634803e-05	88
hsa04150	mTOR signaling pathway	0.0006114137926066184	6.154770701913108e-05	59
hsa05225	Hepatocellular carcinoma	0.0006114137926066184	7.411076274019616e-05	63
hsa05214	Glioma	0.0008561020059955173	0.00012971242515083596	33
hsa05220	Chronic myeloid leukemia	0.00097164552531481	0.00017666282278451092	33
hsa05212	Pancreatic cancer	0.001813741662705709	0.00043275590079873394	32
hsa05222	Small cell lung cancer	0.001813741662705709	0.00045431118829967885	38
hsa04211	Longevity regulating pathway	0.001813741662705709	0.000494656817101557	36
hsa01521	EGFR tyrosine kinase inhibitor resistance	0.003195869923413706	0.0009684454313374866	32
**Common 23 DEGs and 70 DElncRNAs-KEGG Functional Enrichment Analysis**				
**hsaid**	**pathway**	***p*-adjust**	***p*-value**	**Genes**
hsa04010	MAPK signaling pathway	1.22923251613022e-17	1.499064044061244e-19	225
hsa05215	Prostate cancer	8.612608644679657e-14	2.1006362547999165e-15	86
hsa04722	Neurotrophin signaling pathway	7.647697967280332e-13	2.797938280712317e-14	100
hsa04015	Rap1 signaling pathway	2.1920773103682596e-12	1.0693060050576875e-13	165
hsa04152	AMPK signaling pathway	2.4298777304097346e-12	1.4816327624449603e-13	101
hsa05220	Chronic myeloid leukemia	9.72383680842217e-12	7.11500254274793e-13	68
hsa04520	Adherens junction	1.0075525944866993e-11	8.601058733423044e-13	65
hsa05165	Human papillomavirus infection	1.9429917103537356e-11	1.8956016686377907e-12	242
hsa04218	Cellular senescence	2.3876880558830318e-11	2.6206332320667423e-12	124
hsa01522	Endocrine resistance	2.6522564008199485e-11	3.2344590253901814e-12	82
