# Supplementary figures and images for: Comparative Transcriptomic Analyses of Peripheral Blood Mononuclear Cells of COVID-19 Patients without Pneumonia and with Severe Pneumonia in the First Year of Follow-Up
Source: Viruses. 2024 Jul 28;16(8):1211. doi: 10.3390/v16081211 (PMC11358892; doi:10.3390/v16081211)

**a****All DElncRNAs**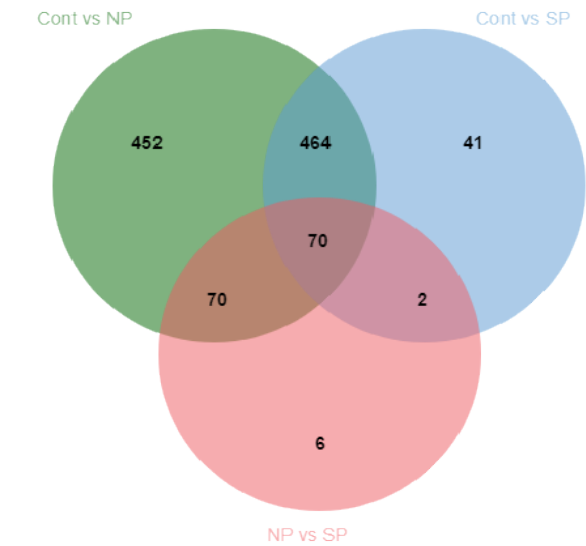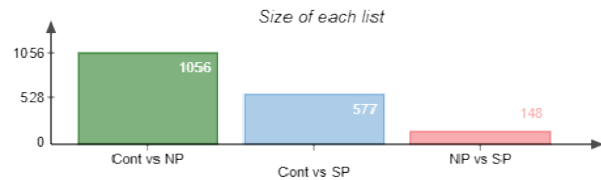**b****Up DElncRNAs**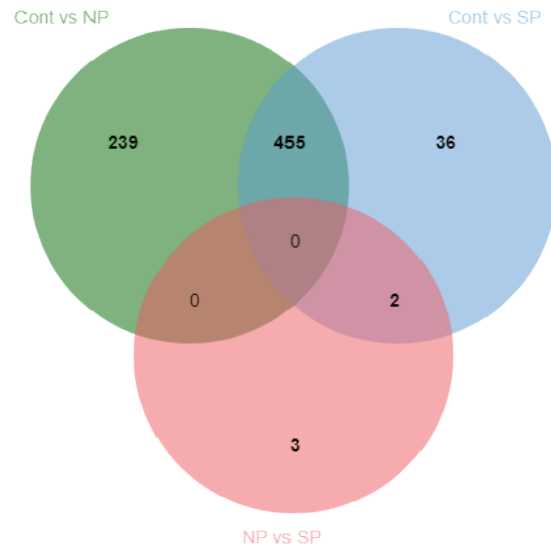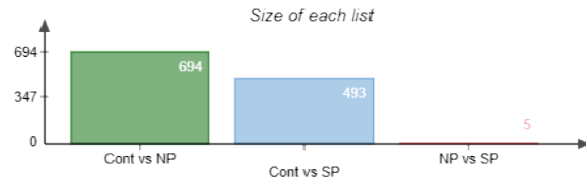**c****Down DElncRNAs**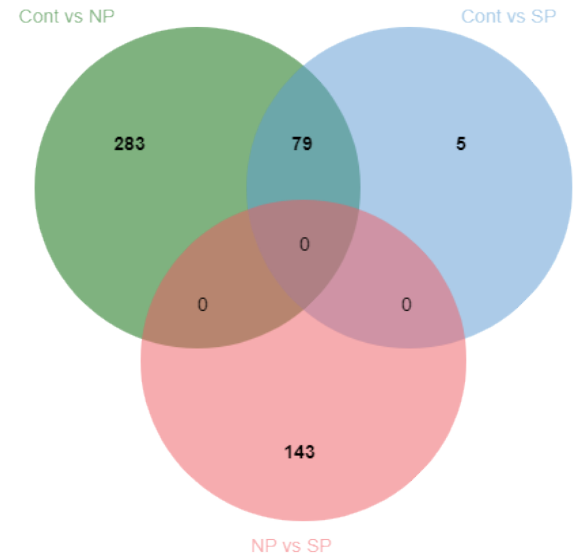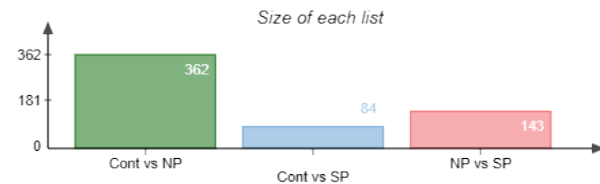

Supplement: Supplementary file 1 [file viruses-16-01211-s001.zip › Supplementary Figure S1.pdf]

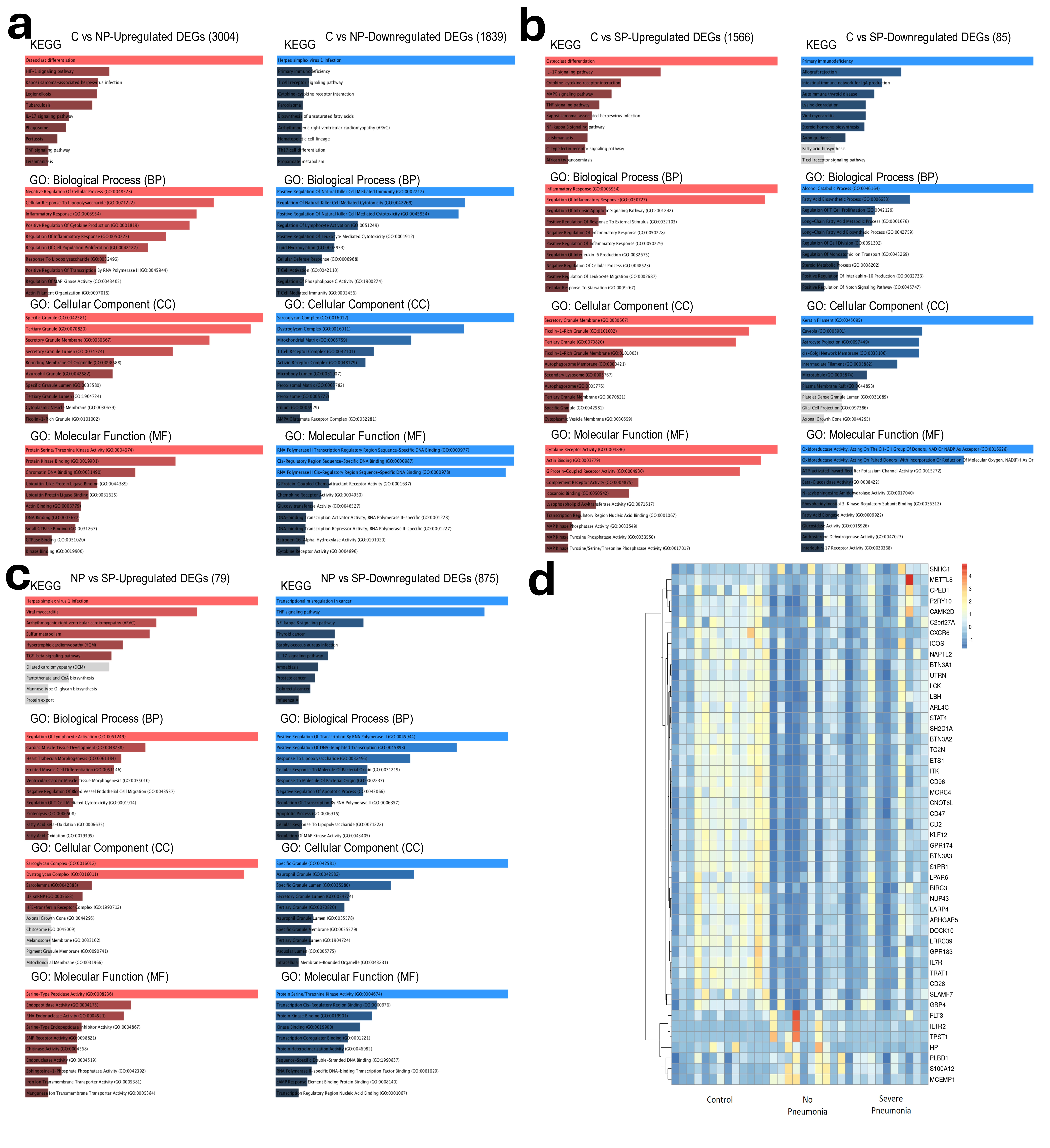

Supplement: Supplementary file 1 [file viruses-16-01211-s001.zip › Supplementary Figure S2.jpg]
